# Interactions between circRNAs and miR-141 in Cancer: From Pathogenesis to Diagnosis and Therapy

**DOI:** 10.3390/ijms241411861

**Published:** 2023-07-24

**Authors:** Małgorzata Guz, Witold Jeleniewicz, Marek Cybulski

**Affiliations:** Department of Biochemistry and Molecular Biology, Medical University of Lublin, 20-093 Lublin, Poland

**Keywords:** ncRNA, microRNA, miR-141, circRNA, cancer, diagnosis, therapy, circRNA-miRNA-mRNA regulatory network

## Abstract

The function of non-coding RNAs (ncRNAs) in the pathogenesis and development of cancer is indisputable. Molecular mechanisms underlying carcinogenesis involve the aberrant expression of ncRNAs, including circular RNAs (circRNAs), and microRNAs (miRNAs). CircRNAs are a class of single-stranded, covalently closed RNAs responsible for maintaining cellular homeostasis through their diverse functions. As a part of the competing endogenous RNA (ceRNAs) network, they play a central role in the regulation of accessibility of miRNAs to their mRNA targets. The interplay between these molecular players is based on the primary role of circRNAs that act as miRNAs sponges, and the circRNA/miRNA imbalance plays a central role in different pathologies including cancer. Herein, we present the latest state of knowledge about interactions between circRNAs and miR-141, a well-known member of the miR-200 family, in malignant transformation, with emphasis on the biological role of circRNA/miR-141/mRNA networks as a future target for novel anti-cancer therapies.

## 1. Introduction

Non-coding RNAs (ncRNAs), including microRNAs (miRNAs) and circular RNAs (circRNAs), function as a key regulators of gene transcription and translation [1]. The deregulated expression of ncRNAs is involved in the development and progression of various types of cancer, since it is known that they are associated with cancer-cell growth, metastasis, and chemoresistance [2].

The well-known members of ncRNAs are miRNAs that are single-stranded RNAs, about 22 nucleotides in length, transcribed by RNA polymerase II or III into primary RNAs (pri-miRNAs) which are processed to precursor miRNAs (pre-miRNA) and ultimately into mature miRNAs [3]. Most miRNAs regulate gene expression through complementary base pairing with target sequences in 3′ untranslated regions (UTRs) of mRNAs; however, it is known that miRNAs are also able to bind to 5′UTRs or even within mRNA coding sequences (CDS) [4]. The interaction between miRNAs and target mRNAs leads to translational repression or mRNA degradation [5]. It was discovered that the expression of a single mRNA may be regulated by multiple miRNAs and one miRNA may regulate multiple genes [6]. These ncRNAs play important roles in cell homeostasis; therefore, any abnormalities in miRNA expression patterns may promote cancer development [2]. MiRNAs may function as oncogenic miRNAs by targeting tumor suppressor genes or tumor suppressor miRNAs that target oncogenes [7]. Due to the stability of cell-free circulating miRNAs in biofluids such as blood, urine, sputum, saliva, and milk, they are considered potential diagnostic biomarkers [7]. Moreover, miRNAs are also considered promising therapeutic targets alone or in combination with other current targeted therapies in different cancers [8,9,10,11].

Circular RNAs (circRNAs) are a single-stranded, covalently closed RNAs without a cap and poly-adenosine tail at the 5′ and 3′ ends, respectively [12]. ncRNAs were discovered over 40 years ago, first in plants and a few years later in humans [13]. Recently, high-throughput RNA sequencing of non-polyadenylated transcriptomes and bioinformatic tools allowed scientists to identify thousands of ubiquitous and abundant circRNAs in all eucaryotes, including plants, fungi, protists, fish, insects, and mammals [14]. CircRNAs are involved in the regulation of gene expression at transcriptional and post-transcriptional levels by acting as miRNA sponges, RNA-binding proteins (RBPs) sponges and some of them can be translated into proteins [15]. Numerous studies have discovered that circRNAs are involved in the development and progression of many different diseases, including cancer [16]. Because of their covalently closed circular structure, circRNAs are resistant to degradation by RNase R exonuclease and can escape RNA turnover which makes them more stable than linear RNAs [17]. This property increases the accumulation of circRNAs in tissues and extracellularly in biofluids such as plasma, saliva, and urine, which allows us to consider them stable biomarkers for a liquid biopsy [16,18]. In addition, circRNAs have become promising targets for anti-cancer therapy, since it has been revealed that they are involved in cancer progression, metastasis, and multidrug resistance [19].

The association between miRNAs and other ncRNAs such as circRNAs in cancer deserves attention, especially in the context of the diagnosis and treatment of this disease. The above findings prompted us to summarize current knowledge about miR-141, a well-known member of miR-200 family, and its relationship with circular RNAs in the pathogenesis, diagnosis, and treatment of different types of human cancers.

## 2. Biogenesis of miR-141, circRNAs and Importance of Their Interactions

The miR-200 family, which includes miR-200a, miR-200b, miR-200c, miR-141, and miR-429, is highly conserved in higher vertebrates and is physiologically expressed in epithelial cells [20]. This family of miRNAs may be divided into two clusters according to the location on the chromosomes [21,22]. The nucleotide sequence of miR-141 is located on chromosome 12p13.31 and it recognizes the nucleotide sequence 5′-AACACUG-3′ on the target mRNAs [23]. Mature miR-141 appears in two different forms: miR-141-3p and miR-141-5p depending on the orientation of the origin strand [21].

In eucaryotes, most precursor messenger RNAs (pre-mRNAs) are composed of coding sequences called exons and non-coding introns [24] that are excised from pre-mRNAs during splicing [25]. Splicing is a highly regulated process that generates a variety of mature spliced transcripts, each one with unique combination of exons [26]. CircRNAs are formed during non-canonical splicing, termed back-splicing, which is catalyzed by spliceosomal machinery or ribozymes from groups I and II [27]. In addition, circRNAs lack both 5′ cap and polyadenylated tail due to their closed covalent structure that differentiates them from linear canonical 5′-3′ mature mRNAs [27]. CircRNAs can arise from introns or exons, leading to the formation of different types of circRNAs: intronic, exon-intron, and exonic circRNAs, generated when a downstream splice donor (3′ splice site) is connected with an upstream splice acceptor (5′ splice site) covalently [27]. These events occur through different models for circRNA biogenesis: circularization mediated by RBP-mediated circularization, intron pairing-driven circularization, and lariat-driven circularization [28]. In the first model of circularization, RBPs interact with the flanking introns and promote the circularization of exons [29]. RBPs bind specifically to each intron on the flanks creating a bridge which brings the splice donor and acceptor sites close enough to create a loop [12]. In the second model, the complementary sequences of the flanking intron pair with each other to bring the splice sites closer to promote circularization [30]. Flanking complementary sequences, for example Alu sequences, play a crucial role in exon circularization, and perfectly matched complementary sequences may promote the expression of circRNAs [31]. In lariat-driven circularization, known as the exon-skipping mechanism, pre-mRNA partially folded during transcription causes the donor site (5′ splicing site) of the upstream intron to close and attack the receptor site (3′ splicing site) of the downstream intron to create circRNAs from the folded region where the remaining exons form a linear mRNA [31].

**Figure 1:**
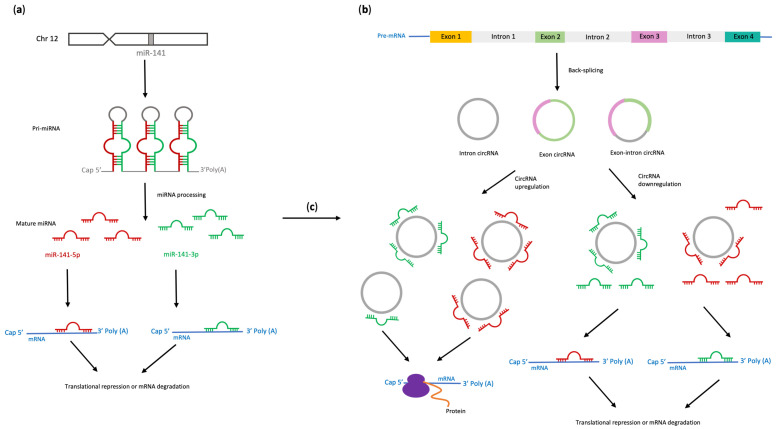
Biogenesis of miRNAs (**a**) and circRNAs (**b**) and their interactions affecting the regulation of gene expression (**c**).

**Figure 2:**
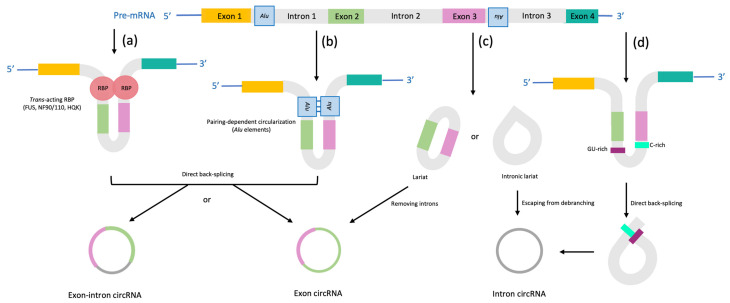
The biogenesis of circRNAs. (**a**) RNA-binding protein-driven circularization (RBPs): RBPs bind to specific flanking sequence motifs of upstream and downstream introns, bring flanking introns closer to each other and causing a formation of exonic-intronic circRNAs or exonic circRNAs. (**b**) The introns flanking inverted repeating elements (such as Alu elements) form exonic circRNAs or exonic-intronic circRNAs by base-pairing, where introns are removed or retained, respectively. (**c**) Exonic circRNAs and intronic circRNAs are created from splicing intermediates (lariat precursors): one mechanism is associated with exon-skipping when one or more exons of the mRNA transcript are skipped during linear splicing, and the second mechanism is based on intronic lariat precursors’ escape from the debranching step of the canonical linear splicing. (**d**) The formation of intronic circRNAs is dependent on 7-nt GU-rich and 11-nt C-rich elements: during direct back-splicing, two elements bind into a lariat-like intermediate which is sufficient to escape from debranching and exonucleolytic degradation. After the trimming of the 3′ tail, intronic circRNAs are generated.

A short summary of miR-141 and circRNA biogenesis with particular emphasis on the role of circRNAs as miR-141 sponges is presented on Figure 1. The different mechanisms of circRNA formation are shown in Figure 2.

## 3. miR-141—An Important Molecular Regulator of EMT in Cancer and EMT-Related Fibrosis in Non-Cancerous Diseases

Epithelial–mesenchymal transition (EMT) is a highly conserved physiological process that controls embryonic development, mesoderm formation, neural crest removal and morphogenesis in other cells [32]. During EMT, epithelial cells lose cell–cell junctions, apical-basal polarity, epithelial markers (E-cadherin, cytokeratins, laminin, MUC-1, collagen type IV-alpha 1, syndecan-1) and acquire motility, a spindle-cell shape and mesenchymal markers (N-cadherin, fibronectin, vimentin, α-smooth muscle actin) [33]. Different cytokines induce EMT: tumor growth factor-β (TGF-β), fibroblast growth factor (FGF), epidermal growth factor (EGF) and hepatocyte growth factor (HGF) [34]. These signals upregulate transcription factors (TFs) including SNAIL, TWIST, and ZEB, which cooperate with miRNAs, for example the miR-200 family [33]. These miRNAs are considered powerful inhibitors of EMT as they target the E-cadherin repressors ZEB1 and ZEB2 [35]. Clustal omega alignments of the human sequences show a significantly higher percentage of identity among mature -3p miRNAs than mature -5p miRNAs of the miR-200 family [36]. Additionally, bioinformatic analysis of transcriptomes from human primary cells indicate that -3p mature miRNAs are highly expressed in epithelial cells in comparison to -5p miRNAs [36]. EMT is a reversible process that plays a crucial role in physiological processes such as wound healing and tissue regeneration, but also in different pathologies such as organ fibrosis and cancer, where the role of miR-141-3p is widely characterized [37]. The influence of lncRNAs on miR-141 expression and their participation in the regulation of EMT during cancer progression is summarized in Table 1. During organ fibrosis, cellular homeostasis is disrupted and extracellular matrix components are excessively deposited. Fibrosis is initiated by injurious stimulation in vital organs or local tissues and can occur, for example, in kidneys (chronic kidney failure), lungs (cystic fibrosis, IPF-idiopathic pulmonary fibrosis), digestive system (Crohn’s disease, liver cirrhosis), heart (myocardial fibrosis, atherosclerosis), peritoneum (peritoneal fibrosis), skin (scleroderma, keloid), and lens (cataract), and ultimately leads to organ dysfunction and failure [38]. In kidneys, the profibrotic cytokine TGF-ß downregulates the expression of miR-141, consequently increasing the expression of HIPK2, and promoting EMT in renal tubular epithelial cells which contributes to renal fibrosis [37]. In renal interstitial fibrosis (RIF), lncRNA TUG1 sponges miR-141-3p and promotes EMT in renal tubular epithelial cells by upregulating the expression of vimentin, α-smooth muscle actin, and β-catenin [39].

In inflammatory bowel disease (IBD), which comprises two major diseases, an ulcerative colitis and Crohn’s disease, downregulation of miR-141-3p leads to the upregulation of its target CXCL12ß [40]. CXCL12ß is a splice variant of the CXCL12 chemokine expressed by intestinal epithelial cells, which participates mainly in the recruitment of memory Th1 cells [41]. Carbohydrate sulfotransferase 15 (CHST15), a type II transmembrane Golgi protein, catalyzes the sulfation of chondroitin sulfate A, which forms chondroitin sulfate E. The CHST15/CS-E axis is involved in fibrosis through two mechanisms, an activation of fibroblasts and the formation of collagen fibrils [42]. CS-E binds different chemokines, including CXCL12 and growth factors, including PDGF and TGF-β, indicating CXCL12 involvement in the proliferation, adhesion, and migration of fibroblasts, which are associated with the migration of these cells into the sites of bowel injury [42].

In lungs, ZEB1 antisense RNA 1 (ZEB1-AS1) lncRNA acting as miR-141-3p sponge downregulates miR-141-3p levels, which increases the expression of miR-141-3p target ZEB1 in lung tissues of IPF and in TGF-ß1-stimulated alveolar type II epithelial cells, which promotes pulmonary fibrosis [43].

It was suggested that EMT may contribute to fibrosis and airway remodeling in asthma [44]. Epithelial cells secrete mucus as a kind of physical barrier between the external environment and internal milieu. This barrier is composed of mucociliary escalators which assist with trapping and removing inhaled foreign particles from the airways, secrete antimicrobial products responsible for killing inhaled pathogens, and form intracellular adherens and tight junctions that regulate epithelial paracellular permeability [45]. MiR-141 expression is high in human airway epithelial cells and lower levels of it are observed in patients with asthma [46]. It was shown that the downregulation of miR-141, using CRISPR/Cas9 gene editing in vitro and antagomir targeting miR-141-3p in vivo, represses the IL-13-induced production of mucus in human bronchial epithelial cells and in an animal model of allergen-induced asthma. Given the important pathogenic role of mucus overproduction in chronic asthma, and the lack of drugs that specifically target the production of mucus in the airways, miR-141 and its target genes may become valuable therapeutic targets in T2-high asthma [46].

Fibrosis is present in all forms of endometriosis and it contributes to classic symptoms related to this disease, such as pain and infertility [47]. Two stimulating signals, hypoxia and estrogen, may activate EMT in endometriosis through TGF-ß and Wnt pathways, leading to cell proliferation and migration [48]. The downregulation of miR-141-3p by other ncRNAs including circRNA promotes EMT and endometriosis progression [49]. Besides its regulatory role in endometriosis, miR-141-3p stimulates the TGF-β2/SMAD2 signaling pathway to promote wound healing. The interplay between lncRNA MALAT1 and miR-141-3p regulates the expression of ZNF17, an oncogenic protein which directly binds to the promoter of TGF-ß. The inhibition of miR-141-3p expression by MALAT1 enhances the activity of TGF-β2/SMAD signaling and stimulates the wound-healing process [50].

Targeting EMT is seen as a novel therapeutic approach for fibrosis-related eye diseases such as the degeneration of the macula or glaucoma-surgery-related fibrosis [51]. The methylation of DNA bases plays an important role in the epigenetic regulation of gene expression, including the modulation of miR-141 expression in normal and cancer cells [52]. Since it is known that miR-141 is involved in EMT, the aberrant methylation of CpG islands near the start of transcription of miR-141/200c cluster is associated with their inappropriate silencing in neoplastic cells and phenotypic conversion in normal cells [52]. The DNA demethylating agent, 5-Azacytidine (5-AzaC), inhibits the transition of human conjunctival epithelial cells towards a mesenchymal phenotype and induces the expression of the miR-200 cluster, including miR-141, suggesting the potential for the invention of epigenetics-based strategies for the treatment of diseases associated with EMT [51].

**Table 1:** The influence of lncRNAs on miR-141 expression and their participation in the regulation of EMT during cancer progression.

Cancer Type	ncRNA	Upregulated miR-141-3p Targets	Biological Effects of miR-141-3p and Its Targets	References
**Gastric cancer (GC)**	lncRNA H19	ZEB1	Inhibition of miR-141increases cell proliferation and invasion, and reduces apoptosis	[53]
	lncRNA MAGI2-AS3	ZEB1	Sponging of miR-141/200a by lncRNA MAGI2-AS3 maintains ZEB1 overexpression and promotes GC progression through increased cell migration and invasion	[54]
		ZEB2	Overexpression of miR-141 reverses EMT in human scirrhous GC and reduces invasion and peritoneal dissemination in nude mice orthotopic tumor model	[55]
			Knock-out of ebv-miRNA (Bart9) increases expression of miR-141, CDH1, and inhibits proliferation and invasion of EBV-associated GC cells	[56]
**Pancreatic cancer (PaC)**		TM4SF1	Overexpression of TM4SF1 promotes PaC cells, EMT, and angiogenesis in vitro and in vivo via Akt signaling pathway	[57]
		NRP1	NRP1 is highly expressed in PaC tissues and promotes proliferation and migration of PaC cells by activation of EMT via TGF-β signaling pathway	[58]
		ZEB1	Downregulation of eIF4E increases expression of ZEB1 and vimentin, and downregulates CDH1 through repression of miR-200c and miR-141	[59]
		ZEB-1, TWIST-1	Overexpression of miR-141 inhibits PDAC cell proliferation, migration, invasiveness, and chemoresistance in vitro. Overexpression of miR-141 and miR-720 downregulates ZEB1 and TWIST-1 leading to upregulation of CDH1 and decreased expression of MAP4K4 resulting in lower phosphorylation of JNK	[60]
**Colorectal cancer (CRC)**		EGFR	miR-141-3p increases cetuximab sensitivity and cetuximab-induced apoptosis, decreases EMT marker expression, suppresses proliferation, migration, and invasion in CRC cells by targeting EGFR pathway	[61]
		ZEB1	Lower expression of miR-141 in oxaliplatin-resistant SW620 cells is associated with increased expression of ZEB1 and vimentin, and higher migration and invasion	[62]
		ZEB1, ZEB2	Knockdown of Ascl2 TF increases miR-141 expression, downregulatesZEB1/ZEB2 and can reverse EMT in vitro	[63]
		ZEB2	MiR-141 and CDH1 are downregulated in poorly differentiated clusters and tumor budding (TB), while ZEB2 is upregulated in TB in CRC	[64]
**Non-small cell lung cancer (NSCLC)**			Overexpression of miR-141 reduces KLF6 expression and increases the secretion of VEGFA in vitro and is related to higher microvessel density in cancer samples	[65]
		PHLPP1, PHLPP2	Overexpression of miR-141 promotes proliferation of NSCLC cells in vitro and cancer growth in vivo through downregulation of PI3K/AKT signaling antagonists, protein phosphatases PHLPP1 and PHLPP2	[66]
	lncRNA XIST	ZEB2	Upregulated lncRNA XIST in metastatic NSCLC tissues decreases miR-141 expression leading to enhanced expression of ZEB2, induction of TGF-β-induced EMT, increased invasion and metastasis.	[67]
		ZEB1	Overexpression of miR-141 downregulates ZEB1, increases expression of CDH1, enhances sensitivity of NSCLC cells to nintedanib and reverses the resistance of nintedanib-resistant NSCLC cells to nintedanib	[68]
	lncRNA FAM83A-AS1		Increased FAM83A-AS1 stimulates EMT, growth, migration, and invasion of LUAD cells through sponging miR-141-3p	[69]
**CRC** **NSCLC**	lncRNA LINC01296	ZEB1, ZEB2	High expression of LINC01296 in CRC and NSCLC cells upregulates ZEB1/ZEB2 by acting as miR-141-3p sponge to promote tumor EMT, invasion, and migration.	[70]
**Breast cancer (BC)**		Sec23A	miR-141 mimics delivered by chitosan nanoplexes to breast cancer cells diminish VEGF, metastasis, invasion, EMT and increase apoptosis	[71]
		ZEB1, ZEB2	PELP1 recruits HDAC2 to repress miR-141 promoter which induces EMT, promotes migration and invasion of BC cells, and stimulates BC growth and metastasis in vivo	[72]
	lnc-ATB	ZEB1, ZEB2	High expression of lnc-ATB in BC cells and tissues decreases miR-141-3p expression to upregulate ZEB1 and ZEB2, stimulate EMT, cell migration and invasion.	[73]
			MiR-141 expression increases after treatment with PARP-1 inhibitor together with gemcitabine which suppresses EMT in TNBC with BRCA1 mutation in vitro	[74]
**Prostate cancer (PC)**		TRAF5, TRAF6	Upregulation of miR-141-3p suppresses EMT, migration and invasion of PC cells and reduces bone metastasis in vivo via inhibition of NF-κB signaling; MiR-141-3p decreases in bone metastatic PC tissues (compared to non-bone metastatic PC); low miR-141-3p correlates with higher PSA level, Gleason grade (differentiation) and bone metastasis status	[75]
**BC and PC**			Aberrant methylation of miR-141 CpG islands is associated with miR-141 silencing suggesting an important role for epigenetic mechanisms in the regulation of EMT	[52]
**Renal cell carcinoma (RCC)**	lncRNA CDKN2B-AS1	CDKN2B-AS1, CCND1, CCND2,	Overexpression of CDKN2B-AS1 in RCC downregulates miR-141, which increases CCND1/CCND2 expression, stimulates proliferation, EMT, clonogenicity, invasion, migration, inhibits apoptosis in vitro and enhances tumor growth in vivo	[76]
		ZEB2	Honokiol, a biphenolic compound isolated from *Magnolia* spp. bark, upregulates miR-141 which suppresses expression of ZEB2, reverses EMT, inhibits formation of tumorspheres by cancer stem cells, and decreases proliferation, migration and invasion of RCC and tumor growth in vivo	[77]
**Diffuse large B-cell lymphoma (DLBCL)**	lncRNA LINC01857	MAP4K4	Upregulation of LINC01857 in DLBCL tissues and cells promotes proliferation, cell cycle, but suppresses apoptosis in DLBCL cells by sponging miR-141-3p, which decreases MAP4K4 and activates EMT and PI3K/mTOR pathway.	[78]
**Head and neck squamous cell carcinoma (HNSCC)**		ZEB1, ZEB2	Enforced expression of miR-141 downregulates ZEB1/ZEB2, increases CDH1 expression, and reduces migration of HNSCC cells	[79]
**Ovarian cancer (OC)**		ZEB	miR-141 mimic downregulates ZEB, upregulates CDH1, inhibits EMT and cell proliferation, decreases migration and invasion in vitro	[80]
		ZEB1, ZEB2, SNAl2	Stable inhibition of miR-141 upregulates expression of ZEB1, ZEB2, SNAl2, vimentin and fibronectin, but downregulates CDH1 and decreases sensitivity of OC cells to paclitaxel and carboplatin	[81]
**Laryngeal cancer (LC)**		HOXC6	Overexpression of miR-141 downregulates HOXC6, inhibits TGF-β signaling, represses EMT, migration, viability, and invasion of LC cells and decreases tumor growth and metastasis to lymph nodes in vivo	[82]
**Nasopharyngeal carcinoma (NPC)**		DLC1	Overexpression of miR-141-3p promotes cell proliferation, migration, invasion, and EMT in NPC cells by targeting DLC1 and activation of mTOR signaling pathway	[83]
**Hepatocellular carcinoma (HCC)**		GP73	Overexpression of miR-141-3p inhibits EMT, proliferation, invasion and migration of HCC cells in vitro, and tumor growth and lung metastasis in vivo	[84]
		ZEB1	Downregulation of miR-141 in HCC with bile duct thrombus is associated with overexpression of ZEB1, TWIST, TGFβRII, vimentin, IL-6, Bmi1 and reduced level of IGFBP-4	[85]
		ZEB1	Increased expression of ELF3 TF downregulates miR-141-3p to stimulate ZEB1 expression, EMT, cell proliferation, migration and invasion in vitro and metastasis in vivo	[86]
**Endometrial cancer (EC)**		ZEB1	Lower expression of miR-141 in epoxomicin-resistant EC cells is associated with the upregulation of ZEB1, downregulation of CDH1 and induction of EMT.	[87]
**Osteosarcoma**		AUF1	miR-141 inhibits proliferation, invasion, and migration of osteosarcoma cells, and suppresses EMT through repression of RNA-binding protein AUF1	[88]

**Abbreviations:** lncRNA H19—long noncoding RNA encoded by the H19 gene (H19 imprinted maternally expressed transcript); lncRNA MAGI2-AS3—long noncoding RNA encoded by MAGI2 Antisense RNA 3 gene; lncRNA XIST—long noncoding RNA X-inactive specific transcript; lncRNA FAM83A-AS1—long noncoding RNA FAM83A antisense RNA 1; lncRNA LINC01296—long intergenic non-protein coding RNA1296; lnc-ATB—long noncoding RNA activated by transforming growth factor-beta; lncRNA CDKN2B-AS1—cyclin-dependent kinase inhibitor 2B antisense noncoding RNA; lncRNA LINC01857—long intergenic non-protein coding RNA 1857; ZEB1—zinc finger E-box binding homeobox 1; TM4SF1—transmembrane 4 L six family member 1; NRP1—neuropilin 1; TGF-β—transforming growth factor- β; eIF4E—eukaryotic translation initiation factor 4E; TWIST-1—twist basic helix-loop-helix transcription factor 1; PDAC—pancreatic ductal adenocarcinoma; MAP4K4—mitogen-activated protein kinase kinase kinase kinase 4; JNK—c-Jun N-terminal kinase; EGFR—epidermal growth factor receptor; ZEB2—zinc finger E-box binding homeobox 2; TF—transcription factor; Ascl2—achaete-scute family BHLH transcription factor 2; CDH1—cadherin 1; KLF6—Krüppel-like factor 6; PHLPP1—PH domain leucine-rich-repeats protein phosphatase 1; PHLPP2—PH domain leucine-rich-repeats protein phosphatase 2; LUAD—lung adenocarcinoma; Sec23A—core component of coat protein-complex II (COPII); VEGF—vascular endothelial growth factor; PELP1—proline, glutamic acid and leucine rich protein 1; PARP-1—Poly[ADP-ribose] polymerase-1; PI3K/Akt—phosphatidylinositol 3-kinase(PI3K)/protein kinase B (Akt); TNBC—triple-negative breast cancer; BRCA1—BRCA1 DNA repair associated; TRAF5—tumor necrosis factor receptor-associated factor 5; TRAF6—tumor necrosis factor receptor-associated factor 6; NF-κB—nuclear factor kappa-light-chain-enhancer of activated B cells; PSA—prostate specific antigen; mTOR—mechanistic target of rapamycin; SNAl2—snail family transcriptional repressor 2; HOXC6—homeobox C6; DLC1—deleted in liver cancer 1/StAR-related lipid transfer protein 12; GP73—Golgi protein 73; TGFβRII—transforming growth factor beta receptor II; IL-6—interleukin 6; Bmi1—B-lymphoma Moloney murine leukemia virus insertion region 1; IGFBP-4—insulin-like growth factor binding protein-4; ELF3—E74-like ETS transcription factor 3; AUF1—AU-rich element RNA-binding factor 1.

## 4. circRNAs as Sponges for miR-141 in Different Types of Human Cancers

One of the best-understood biological functions of circRNAs is to control translation by acting as sponges for miRNAs, leading to the upregulation of corresponding mRNA targets [89]. A single circRNA can harbor one or more binding sites for an miRNA [90] and therefore circRNAs expressed in mammalian cells are involved in the regulation of various physiological processes [91]. The deregulated expression of circRNAs is associated with many diseases including cancer [92]. The over-expression of tumor-suppressive circRNAs that bind oncogenic miRNAs can upregulate tumor-suppressor mRNAs and therefore inhibit cancer-cell proliferation, stimulate apoptosis, and reduce EMT [93]. On the other hand, cancer-promoting events occur when oncogenic circRNAs are highly expressed as they bind tumor-suppressive miRNAs, which upregulate the expression of oncogenic mRNAs, stimulate carcinogenesis, and promote cancer progression [93]. The interactions between various circRNAs and miR-141 discussed below are summarized in Table 2.

### 4.1. Breast Cancer

Globally, about 19 million new cancer cases occurred in 2020, and female breast cancer (BC) surpassed lung cancer as the most frequently diagnosed cancer, with 2.3 million new cases [109]. BC is a heterogenous disease influenced by various cancer-cell intrinsic parameters, such as the genetic profile, stemness, proliferation, intrinsic cell plasticity, capacity for migration, invasion, and the interplay between genome, epigenome, transcriptome and proteome, as well as tumor microenvironmental factors, for example hypoxia, the degree of vascularization, interaction between cancer cells and stromal tumor cells, and the contribution of tumor-infiltrating cells of the innate and the adaptive immune systems [110]. All these factors that constitute “intertumoral heterogeneity”, significantly affect treatment options and patient prognosis. Heterogeneity in the expression of estrogen receptor (ER), progesterone receptor (PR) and human epidermal growth factor 2 (HER2) is assessed by immunohistological staining in all invasive BCs [111], and about 70% of advanced BCs are hormone-receptor positive, defined by the expression of ER, PR, or both [112]. The triple-negative breast cancer (TNBC) which accounts for 10–15% of all BC cases is characterized by the loss of expression of HER2 and both hormonal receptors [113]. TNBC is an aggressive subtype of BC with a high relapse rate, poor prognosis and limited treatment options [114]. The most recommended treatments for metastatic TNBC patients are doxorubicin, liposomal doxorubicin, capecitabine, vinorelbine, gemcitabine, eribulin, olaparib, cisplatin, carboplatin, talazoparib, paclitaxel, and atezolizumab combined with nab-paclitaxel [114]. The high recurrence rate of TNBC is due to its chemoresistance which involves ncRNAs (miRNAs, lncRNAs, and circRNAs) [115]. In vitro studies showed the overexpression of hsa_circ_0009362 (circGNB1), generated from two exons, 2 and 3, of the *GNB1* gene with no intron, in TNBC cell lines [94]. The upregulation of this ncRNA in TNBC tissues was associated with worse clinical features, shorter overall survival (OS) and disease-free survival (DFS), and circGNB1 was revealed as an independent risk factor for TNBC patients [94]. Moreover, it was discovered that CircGNB1 sponges miR-141-5p and upregulates insulin-like growth factor 1 receptor (IGF1R), which promotes proliferation and migration of TNBC cells, and increases tumor growth and the number of lung metastases in a mouse xenograft model [94]. The stimulation of IGF signaling pathways enhances the growth, drug resistance, and metastasis in many types of human cancers and IGFR1 expression is elevated in about 50% of BCs [116]. As the knockout of circGNB1 decreases IGFR1 expression, which suppresses the growth and metastasis of TNBC, circGNB1 may become a novel therapeutic target and biomarker of TNBC in the near future [94].

Another study discovered that hsa_circ_0075943 is overexpressed in BC cell lines and tissue specimens [95]. Hsa_circ_0075943 has multiple binding sites for miR-141-3p, which increases AK2 mRNA and protein levels in BC cells and tumor samples [95]. AK2 is an isoform of adenylate kinase, a critical enzyme in the cellular homeostasis of adenine nucleotides, which is involved in malignant transformation by the regulation of metabolism of cancer cells, metabolic signaling, invasion and migration [117]. The knock-out of hsa_circ_0075943 was able to inhibit the malignant behavior of BC by increasing the miR-141-3p level, suppressing the growth of cancer cells and inducing apoptosis, which may be crucial in the development of novel therapies for this cancer [95].

### 4.2. Lung Cancer

Lung cancer (LC) is the second most common cancer worldwide, but it is still the leading cause of cancer-related death, with an estimated 1.8 million deaths in 2020 [109]. LC is divided into two groups, non-small-cell lung cancer (NSCLC) and small-cell lung cancer (SCLC). Histological studies revealed that SCLC arises from small neuroendocrine cells of the basal bronchial epithelium and it has two subtypes, pure or combined with NSCLC [118]. NSCLC is divided into large-cell carcinoma (LCC), adenocarcinoma, and squamous cell carcinoma (SCC) [118]. Adenocarcinoma is commonly caused by mutations of *STK11, EGFR, KRAS, ALK*, and the amplification of *MET* [119]. SCC is commonly driven by amplifications of EGFR, phosphatidylinositol-4,5-bisphosphate 3-kinase catalytic subunit alpha (PIK3CA), and MET [119]. SCLC is commonly caused by MET mutations and PIK3CA amplification [119]. Additionally, other abnormalities such as tumor protein p53 (TP53) mutations are highly found throughout all the aforementioned types of lung cancers [119]. Abnormal expression of ncRNAs was noticed in lung cancers and their involvement in lung tumorigenesis and metastasis was confirmed. For example, hsa_circRNA_102442 (circKEAP1) derived from exon 2 of the *KEAP1* gene is downregulated in lung adenocarcinoma tissues in comparison to adjacent normal tissues [96]. In vivo studies showed that circKEAP1 inhibits tumor growth by sponging miR-141-3p as a result of binding of this miRNA at two sites [96]. Interestingly, miR-141-3p binds to 3′-UTR *KEAP1* mRNA and suppresses Kelch-like ECH-associated protein 1 (KEAP1) at the protein level [96]. Under unstressed conditions, KEAP1 acts as a substrate adaptor protein for an E3 ubiquitin ligase complex (Cullin-3/Rbx-1) that ubiquitinates NRF2 protein, a basic-region leucine zipper TF, leading to its proteasomal degradation [120]. Under oxidative stress, NRF2 dissociates from the KEAP1-Cullin3 complex and translocates to the nucleus, where it activates the expression of cytoprotective genes implicated in protection against cancer [121]. Interestingly, the suppression of miR-141-3p by circKEAP1 increases KEAP1 protein levels, which decreases the NRF2 protein level and inhibits cancer growth. These findings suggest that the circKEAP1/miR-141-3p/KEAP1 axis may become a potential target for novel treatments of lung adenocarcinoma [96].

### 4.3. Gastric Cancer

Gastric cancer (GC) is still an important cancer globally with over one million new cases and almost eight hundred thousand deaths in 2020, ranking this neoplasm fifth for incidence and fourth for mortality worldwide [109]. Programmed-death ligand 1 (PD-L1) with its receptor programmed cell-death 1 (PD-1) are crucial proteins of immune inhibitory checkpoints that help to maintain immune homeostasis, play a key role in chronic infections, and contribute to cancer immune escape [122,123]. PD-L1, also known as CD274 or B7H1, is highly expressed on the cell surface of antigen-presenting cells and cancer cells, whereas PD-1 (CD279) is expressed on the surface of immune cells such as T-cells, B-cells, natural killer (NK) cells, macrophages, monocytes, and dendritic cells [124]. In the tumor microenvironment, PD-L1 expressed on cancer cells interacts with PD-1 on T-cells which inhibits the function of the latter cells by reducing their proliferation, inhibiting cytokine secretion, and inducing their apoptosis [125,126]. Various signals modulate the PD-1/PDL1 axis, including the JAK-STAT pathway, which participates in cancer-driven immune escape [126]. It was reported that infection of stomach with Helicobacter pylori, which is one of the major risk factors associated with the development of GC, induces the expression of PD-L1 and STAT1 in gastric epithelial cells that promotes immune escape and allows the progression of premalignant lesions to GC [127]. PD-L1 expression was found to be a prognostic marker for GC, which correlates with cancer diameter and depth of penetration, and is associated with molecular features, such as Epstein–Barr virus (EBV) infection and microsatellite instability [128,129]. Therapies that prevent recognition between PD-1 and PD-L1 restore the normal function of T-cells and belong to highly promising immunotherapy approaches [125]. Monoclonal antibody-based therapies targeting PD-1 (nivolumab and pembrolizumab), PD-L1 (avelumab, atezolizumab, durvalumab) and cytotoxic T-lymphocyte antigen 4 (ipilimumab) are promising options of treatment for patients with advanced GC, and likewise the combination of these immune checkpoint inhibitors with chemotherapy, which is the subject of clinical trials [130]. It was reported that hsa_circ_0008583 derived from exons 13, 14, 15, 16 of DLG1 gene is upregulated in distant metastatic lesions and primary GC tissues from patients undergoing anti-PD-1 therapy [97]. Moreover, ectopic expression of this circRNA enhances proliferation, migration, invasion, and immune evasion in vitro, as well as GC progression and metastasis in immunocompetent mice [97]. Mechanistically, circDLG1 interacted with miR-141-3p, resulting in the upregulation of the miR-141-3p target gene C-X-C motif chemokine 12 (CXCL12), which induced infiltration of myeloid-derived suppressor cells (MDSCs) to impair the function of CD8^+^ T cells and promoted GC progression [97]. CXCL12 is a chemokine that binds to receptors CXCR4 and CXCR7 and regulates tumor metastasis through different mechanisms, such as facilitating proliferation, angiogenesis, and colonization, affecting endothelial adhesion, eruption from blood vessel and evasion from host immune response by activation of key pro-survival pathways, such as the previously mentioned JAK/STAT signaling and ERK/MAPK and PI3K/AKT/mTOR signaling pathways [131]. Additionally, chemokine CXCL12 regulates cell adhesion, promotes communication between neoplastic cells and non-malignant cells in the tumor microenvironment, and activates tumor-infiltrating immune cells, such as macrophages and neutrophils [131,132]. These findings suggest that the circDLG1/miR-141-3p/CXCL12 axis may become a novel target for GC therapies.

### 4.4. Prostate Cancer

Prostate cancer (PC) is the second most frequently diagnosed cancer after lung cancer in men and the fifth most common cause of cancer deaths worldwide in 2020 [109]. Numerous epidemiological studies have revealed an increased risk of PC in patients with prostatitis and benign prostatic hyperplasia (BPH) [133]. The latest study showed that BPH is genetically related to PC and both diseases share common inherited polygenic risk [134,135]. Common features of both pathologies are hormone-dependent growth and a pharmacological effect for anti-androgen drugs [135]. Genetic and epigenetic molecular patterns that promote prostate carcinogenesis are the loss of *PTEN*, amplification and overexpression of *MYC*, fusion between *TMPRSS2* and *ERG*, inactivation of *RB* and *P53,* and dysregulation of Krüppel-like factor 5 (KLF5) [136]. KLF5, a zinc-finger TF, regulates the expression of a large number of genes involved in diverse processes including cell proliferation, apoptosis, angiogenesis, autophagy, stemness, migration, and EMT [137]. In PC, KLF5 binds to the androgen receptor (*AR*) gene promoter and enhances its transcription. Furthermore, the interaction of KLF5 with AR increases the transcription of *MYC*, *CCND1,* and *PSA* genes [137]. On the other hand, the interaction of KLF5 with estrogen receptor β (ERβ) promotes the expression of *FOXO1* and suppresses PC growth in vivo [137]. KLF5 is frequently downregulated and deleted in PC, which is associated with the aggressiveness of this cancer [138]. KLF5 may act as a suppressor of PC invasion and metastasis by inhibiting IGF1 transcription and STAT3 activity [138]. Additionally, the downregulation of KLF5 was associated with the progression of PC, poor patient prognosis, and desensitized castration-resistant PC cells to docetaxel by inducing cell autophagy [139]. Apart from the low expression of KLF5 in PC, downregulation of circRNA hsa_circ_0001206 arising from the second exon of *CRKL* gene (circCRKL) and high expression of miR-141 were detected in PC cells and tissues [98]. circCRKL was revealed to act as a tumor suppressor in PC by binding miR-141, leading to the upregulation of miR-141 target KLF5, repressed cell cycle, invasion, migration, boosted apoptosis of PC cells, and reduced tumor progression in vivo [98]. A recent study discovered that a novel circular RNA, circSOBP derived from the exons 2 and 3 of the sine oculis binding protein homolog (*SOBP*) gene, is downregulated in PC tissues compared to adjacent noncancerous prostate tissues [99]. CircSOBP sponges miR-141-3p and upregulates MYPT1 expression, a molecular target of miR-141-3p [99]. In turn, MYPT1 dephosphorylates myosin light chain 2 (p-MLC2), inhibits migration and invasion of PC cells by the regulation of their ameboid migration [99]. Activation of myosin II by the phosphorylation of its MLC2 at Ser19 allows it to interact with actin and generates contractile forces crucial for contraction, migration, cytokinesis, and membrane blebbing [140]. Overexpression of circSOBP inhibits the migration and invasion of PC cells in vitro and metastasis in vivo, and the molecular mechanism described above may become a promising target for a novel therapy for patients with PC [99].

Dose-escalated external beam radiation therapy (EBRT) and EBRT plus high-dose rate brachytherapy (HDR-BT) are well-established recommended treatment options for localized PC [141]. It was discovered that circRNAs can improve the effectiveness of radiation therapy in PC through miR-141-3p sponging and the regulation of TR4/QKI/circZEB1/miR-141-3p/ZEB1 axis [100]. Testicular receptors, including testicular receptor 4 (TR4), are classified as orphan receptors and they play important functions in PC progression, such as migration and invasion as well as resistance to radiotherapy and chemotherapy [142]. TR4 can be trans-activated by phosphorylation, sumoylation, acetylation, and it can be modulated by the metformin through the activation of AMPK and phosphorylation of TR4, while the suppression of trans-activation of TR4 can occur via increased acetylation in its DNA binding domain [143]. The suppression of TR4 promotes radiosensitivity and better suppresses the progression of PC through the modulation of the protein quaking (QKI)/circZEB1/miR-141-3p/ZEB1 signaling pathway. CircZEB1 is generated by back-splicing from the exons 2, 3, and 4 of the *ZEB1* gene [100]. TR4 increases circZEB1 expression through enhanced transcription of RNA-binding protein QKI [100], and circZEB1 and 3′UTR of ZEB1 mRNA can bind miR-141-3p, thus the sponging of miR-141-3p by circZEB increases the expression of *ZEB1* gene [100]. TR4/QKI/circZEB1/miR-141-3p/ZEB1 axis can be activated after administration of metformin and ionizing radiation which promotes radiosensitivity and suppresses the growth of PC in vivo [100].

### 4.5. Liver Cancer

The most common primary liver cancer is hepatocellular carcinoma (HCC) (75–85% of cases) followed by intrahepatic cholangiocarcinoma (10–15% of cases). Liver cancer was the sixth most commonly diagnosed neoplasm and the third leading cause of cancer-related death globally in 2020 [109]. HCC susceptibility is associated with hepatitis B virus (HBV) and C (HCV) chronic infections, alcohol abuse, smoking, type 2 diabetes, and obesity as a leading cause of non-alcoholic steatohepatitis [144,145]. It is not known if HCC is caused by specific mutations, but increased risk of developing HCC is observed in patients with specific germline DNA polymorphisms in genes such as EGF, INFL3, MBOAT7, MICA, PNPLA3, TTL1, TM6SF2, and in heritable conditions including α_1_-antitripsin deficiency, porphyria cutanea tarda, tyrosinemia, and Wilson’s disease [145]. In addition, analysis of the HCC DNA methylation profile revealed hypomethylated genes encoding matrix metalloproteases—MMP2, MMP9, and MMP12 [101]. Additionally, the deregulated expression of ncRNAs including lncRNAs, circRNAs, and miRNAs contribute to tumorigenesis and progression of this liver cancer [146]. Circular RNA, circ_100338, is upregulated in HCC tissues, and is correlated with metastatic progression and a low cumulative survival rate in patients with HBV-related HCC [102]. MiR-141-3p was discovered as a direct target of circ_100338, and both ncRNAs regulated metastatic potential of liver cancer cells. Moreover, circ_100338 activated the mTOR signaling pathway through circ_100338/miR-141-3p/RHEB axis and its increased expression was associated with poor prognosis for patients with HCC [101,102]. In normal cells, the mTOR signaling pathway is an important regulator of cell growth and division, but in tumor cells abnormal activation of this pathway encourages tumor cells to grow, invade healthy tissues, and metastasize [147]. The upregulation of circ_100338 and sponging of miR-141-3p leads to the upregulation of RHEB mRNA, which is a molecular target of miR-141 and a well-characterized activator of mTOR signaling [101]. A GTP-binding protein RHEB, ubiquitously expressed in all tissues, is anchored to lysosomal membrane and its GTP-bound form activates mTORC1, an important regulator of cell growth and proliferation [148]. mTOR involves two distinct complexes, mTORC1 and mTORC2, that participate in response to growth factors, but generally hyperactivation of the mTORC1 is involved in the regulation of cell proliferation and progression of cancer [148]. The downstream effectors of the mTOR signaling pathway are eukaryotic translation initiation factors (eIFs) which contribute to hallmarks of cancer such as uncontrolled growth, sustained proliferative signaling, replicative immortality, angiogenesis, resistance to apoptosis, invasion, and metastasis [149]. It was shown that eIF5 is regulated by circ_100338/miR-141-3p/RHEB axis and higher expression of RHEB or eIF5 was an indicator of shorter OS in hepatitis B-related HCC [101]. Additionally, the upregulation of circ_100338 increases expression of ZEB1 mRNA, a direct target of miR-141-3p, which increases proliferation of HCC cells both in vitro and in vivo [103]. Piperlongumine, also known as piplartine, a natural alkaloid isolated from long pepper (Piper longum), was able to decrease the expression of circ_100338 and repress proliferation of HCC cells [103]. Piplartine showed antiproliferative activity against other cancer cells without affecting normal cells in vitro and in vivo [150,151,152], and it was discovered that it modulates cell signaling pathways that play crucial roles in the initiation and progression of cancer, such as the PI3K/Akt/mTOR, NF-κB, JAK/STAT3 and ERK signaling pathways [153]. These findings prove that targeting of circRNAs with chemical agents may improve the treatment of different cancers.

Estrogens regulate normal liver metabolism and can influence the progression of HCC by acting through the estrogen receptor ERα to regulate cell invasion [104]. ERα is the predominant type of ERs expressed in both male and female hepatocytes, where it plays important roles in the regulation of glucose and cholesterol homeostasis [154]. ERα acts as a tumor suppressor in the HCC progression through several pathways including STAT3 pathway, lipid-metabolism-related pathways, and ncRNAs [155]. ERα decreases the invasion of HCC cells by the suppression of circ-SMG1.72 transcription through binding to the 5′ promoter region of SMG1 host gene, and HCC patients with higher expression of ERα have higher OS and recurrence-free survival rates [104]. circ-SMG1.72 directly sponges miR-141-3p which inhibits HCC invasion by suppressing the expression of gelsolin mRNA, the member of actin-binding proteins responsible for the modulation of cell motility, morphogenesis, apoptosis, and actin cytoskeletal remodeling [104,156]. Downregulation of circ-SMG1.72 by ERα decreases the sponge function of this circRNA for miR-141-3p that reduces the expression of miR-141-3p target gelsolin and inhibits the invasion of HCC. Therefore, targeting the ERα/circ-SMG1.72/miR-141-3p/gelsolin signaling pathway with small inhibitors may be useful for the suppression of HCC progression [104].

### 4.6. Thyroid Cancer

The incidence of thyroid cancer (TC) has risen over the past four decades worldwide [157] with almost six hundred thousand new cases and over forty thousand deaths in 2020 [109]. The main histologic type of this cancer is papillary TC (PTC) accounting for about 90% of TCs [157]. Other types are follicular TC, Hürthle-cell carcinoma, medullary TC that originates from calcitonin-secreting C cells, and highly aggressive anaplastic TC [157,158]. The highest increases in incidence were observed for small and localized TCs; therefore, the rising incidence trends for TC have been mainly attributed to overdiagnosis, resulting from the use of more sensitive diagnostic tools and imaging. Additionally, the increasing incidence of large and advanced TCs, as well as TC mortality, suggest that etiological factors contribute to increasing incidence of this disease [157,159]. Current diagnostic TC markers include point mutations in the BRAF, HRAS, KRAS, and NRAS genes, rearrangements in PAX8/PPARG and RET genes, and point mutations in the promoter region of the TERT gene [160]. NcRNAs are crucial factors in the tumorigenesis, metastasis, diagnosis, and treatment of TC [161,162]. The comparative analysis of microarray profiles of circRNAs in PTC tissues, benign thyroid lesions, and normal samples revealed the upregulation of 129 and downregulation of 226 circRNAs [105]. Moreover, bioinformatics tools identified interactions between cancer-related miRNAs miR-141-3p/miR-200a-3p and circRNA_100395, and target genes of the hsa_circRNA_100395/miR-141-3p/miR-200a-3p axis such as E2F3, CCND2, CCNG1, CDC25B, GLS, PTEN, and ZFPM2 [105]. These results suggest that dysregulation of circRNAs/miRNA network may play an important role in the pathogenesis of PTC, and several circRNAs may become new biomarkers and potential targets for the diagnosis and treatment of PTC [105].

### 4.7. Bladder Cancer

Bladder cancer (BlC) was the 10th most commonly diagnosed neoplasm worldwide, with about 573,000 new cases and 213,000 deaths in 2020 [109]. BlC remains the most common cancer of the urinary tract, and in 75% of patients it is diagnosed as NMIBlC—non-muscle invasive disease [163]. In the remaining 25% of cases, BlC is identified as MIBCa—muscle-invasive disease, as it invades deeper layers of the bladder and forms metastases [163]. Urine cytology, a simple non-invasive method, combined with cystoscopy, allows for the detection of high-grade lesions (such as carcinoma in situ), but it has poor sensitivity for detecting low-grade BlCs [164]. The heterogeneity of BlC calls for more in-depth molecular characterization, which will help to discover new diagnostic markers and treatment options. In recent years, ncRNAs were found to be linked with BlC occurrence and development [165]. It was found that exosome-derived hsa_circ_0085361 (circTRPS1) sponges miR-141-3p and modulates the expression of glutaminase-1 (GLS1) in BlC cells [106]. GLS1 is a kidney isoform of glutaminase, an enzyme that hydrolyzes glutamine to glutamate, which is often upregulated in quickly growing cancer cells to fuel their rapid proliferation. Except glutaminolysis, GLS1 is involved in the regulation of cellular metabolism, maintaining redox balance and glutathione biosynthesis in cancer cells [166]. Exosomes released from circTRPS1-knock down BlC cells were able to prevent CD8^+^ T-cells from exhaustion in the tumor microenvironment and reduced the malignant phenotype of BlC cells. Taken together, these results suggest that the circTRPS1/miR-141-3p/GSL1 axis may become a new therapeutic target for BlC [106].

### 4.8. Oral Squamous Cell Carcinoma

Cancers of the lip and oral cavity were the 16th most commonly diagnosed neoplasms worldwide, with almost 378,000 new cases and 178,000 deaths in 2020 [109]. The majority (90–95%) of oral cancers (OCs) are oral squamous cell carcinomas (OSCCs) that are characterized by high mortality due to local aggressiveness, metastasis, and recurrence [167]. Alcohol consumption, smoking, tobacco consumption in different forms (areca nut, beeri, pan, betel quid, gutka, mainpuri, khiwam, khaini, naswar, zarda, supari, and an attractive sachet of pan masala), viral infections including human papillomavirus (HPV), herpes simplex virus (HSV), and EBV, are important etiological factors of OSCC [168]. Immunosuppressed patients have increased risk and poorer survival in OSCC as the dysregulation of immunosuppressive genes encoding cytokines, e.g., interferon-γ (IFN-γ), interleukin 2, 4, 6, 15, 18, 23, tumor necrosis factor-α (TNF-α), and chemoattractant chemokines such as CCL-2, CXCL-9, and CXCL-10 contribute to the development of OCs [169]. Additionally, the incidence of OCs is increased in patients with a history of immunosuppression (autoimmune disease, organ transplant, pulmonary disorder, myeloproliferative disorder, hematological malignancy, and HIV infection) [169]. The deregulated expression of ncRNAs participates in OSCC development, progression, and drug resistance [170]. The expression of hsa_circ_0072387 in OSCC tissues was significantly lower than in adjacent normal tissues, as well as in three OSCC cell lines compared to normal oral keratinocytes [107]. The bioinformatic analysis predicted that hsa_circ_0072387 can bind miR-141-3p which may play an important role in OSCC progression by the regulation of different tumor-related signaling pathways, where the most enriched and meaningful was the MAPK pathway [107].

### 4.9. Bone Cancer

Osteosarcoma, a primary bone cancer of mesenchymal origin, occurs in children, young adults and has the highest incidence in the second decade of life (15–19 years) with the second peak at 75–79 years [171,172,173]. Its worldwide incidence is low (3.4 per year per million people), but it is often presented by extreme lung metastases with about 40% 5-year survival [173]. WHO divided osteosarcoma into central, intramedullary, and surface cancers with a number of subtypes in each group [172]. In most cases, osteosarcoma arises sporadically and is characterized by driver mutations in tumor-suppressor genes such as *p53, RECQL4, Rb, BLM* and *WRN* in patients with Li–Fraumeni, Rothmund–Thomson, hereditary retinoblastoma, Bloom or Werner syndromes, respectively [173]. It was proposed that the evaluation of biomarkers characteristic for osteosarcoma cells such as *VIM*, *Ezrin* and *COL5A2* in blood may be used as a diagnostic and prognostic tool for patients with osteosarcoma [174]. ncRNAs were recognized as key molecular players with prognostic, predictive and therapeutic significance for osteosarcoma [175,176,177]. Circ-LRP6 expression was found to be upregulated in osteosarcoma tissues and cell lines in comparison to paracancerous tissues and normal osteoblasts, respectively. Circ-LRP6 sponged miR-141-3p, which resulted in higher expression of two targets of this miRNA, *HDAC4* and *HMGB1*, and promoted the proliferation, migration, and invasion of osteosarcoma cells [108]. These results are consistent with other studies showing that the overexpression of HDAC4 in osteosarcoma cells promotes proliferation, invasion, and inhibits apoptosis [178]. In turn, HMGB1, a DNA chaperone, was connected with the progression of different cancers and apoptosis, chemoresistance, and proliferation of osteosarcoma cells [179,180].

## 5. Summary of the Importance of Interactions between ncRNAs and miR-141 in Cancer

MiR-141, a member of miR-200 family, acts mainly as a tumor suppressor, but it may behave as an oncogene depending on cancer type. Additionally, miR-141 biological function can be inhibited by its interaction with ncRNAs such as circRNAs and lncRNAs.

### 5.1. miR-141 Acting as Tumor Suppressor

The sponging of miR-141-5p by circGNB1 or hsa_cicrc_0075943 in BC promotes proliferation and migration of TNBC cells, inhibits apoptosis, and stimulates cancer growth and metastases in vivo [94,95]. Additionally, lnc-ATB decreases miR-141-3p expression to stimulate EMT, migration, and invasion in BC cells and tissues [73]. The overexpression of circDLG1 in GC cells and tissues inhibits miR-141-3p and impairs the function of CD8^+^ T cells that promote GC progression [97]. Moreover, inhibition of miR-141 by lncRNAs (H19, MAGI2-AS3) in GC increases cell proliferation, migration, and invasion, reduces apoptosis, and induces EMT [53,54,55,56]. In liver cancer, circRNA-100338 inhibits miR-141-3p, which enhances the proliferation of HCC cells both in vitro and in vivo [103], and its increased expression is associated with worse prognoses for patients with HCC [101,102]. Additionally, the downregulation of circRNA-SMG1.72 decreases sponge function of this circRNA for miR-141-3p leading to the inhibition of the invasion of HCC [104]. In bone cancer, circ-LRP6 suppresses miR-141-3p, which promotes proliferation, migration, and invasion of osteosarcoma cells [108]. In lung cancer, lncRNAs XIST and FAM83A-AS1 inhibit miR-141 leading to induction of EMT, increased growth, migration, invasion, and metastasis [67,69]. Similarly, lncRNA LINC01296 promotes EMT, invasion, and migration by sponging miR-141-3p in CRC and NSCLC [70]. Overexpression of lncRNA CDKN2B-AS1 in RCC downregulates miR-141, which stimulates proliferation, EMT, clonogenicity, invasion, migration, inhibits apoptosis in vitro, and enhances tumor growth in vivo [76]. Upregulation of lncRNA LINC01857 in DLBCL tissues and cells promotes proliferation and the cell cycle, but suppresses apoptosis in DLBCL cells by sponging miR-141-3p [78].

### 5.2. miR-141 Acting as an Oncogene

On the other hand, the suppression of miR-141-3p by circKAP1 inhibits LC growth in vivo [96]. The downregulation of miR-141 in PC by circCRKL represses the cell cycle, invasion, migration, induces apoptosis of PC cells, and decreases tumor progression in vivo [98]. Similarly, the suppression of miR-141 by circSOBP inhibits migration and the invasion of PC cells in vitro and metastasis in vivo [99].

Additionally, miR-141 was found to modulate the response of cancer cells to anti-cancer therapy. Sponging of miR-141-3p by circZEB in PC decreases radiosensitivity through TR4-mediated QKI/circZEB1/miR-141-3p/ZEB1 axis [100].

## 6. Conclusions

Cancer is still the leading cause of death worldwide and its incidence increases every year. The scientific world is focused on discovering the mechanisms underlying cancer pathogenesis, which is required for the development of better diagnostic tools and anti-cancer therapies. At present, we know that ncRNAs are involved in carcinogenesis and the interactions between different classes of ncRNAs can disrupt the homeostasis of various cells and tissues. One of the first known and characterized relationships between circRNAs and miRNAs was the interaction of miRNAs with annular RNAs that inhibits the binding of miRNA with mRNA and therefore influences the expression of genes targeted by miRNAs. circRNAs may act as tumor-suppressors or oncogenes depending on the type of miRNA they inhibit. miR-141, a member of the miR-200 family, behaves as a tumor suppressor or oncogene in different cancer types, and the molecular pathways that involve interaction between diverse circRNAs and miR-141 are not fully known yet. Better understanding the complexity of circRNA-miR-141 networks gives a great hope for the translation of theory to clinical applications in cancer diagnosis and treatment in the near future.

In this review, we presented the biogenesis and functions of miR-141, and evaluated the importance of its interactions with circRNAs in cancer and non-cancerous diseases. MiR-141 and associated ncRNAs participate in the regulation of EMT, a process that plays a crucial role in physiological processes (e.g., wound healing, tissue regeneration), and pathologies such as organ fibrosis, endometriosis, and cancer. The sponging of miR-141 by different circRNAs increases the expression of miR-141 target genes, which influences malignant transformation, apoptosis, cell proliferation, migration, invasion, metastasis, immune evasion, and response to therapy in different human cancers. A better understanding of the biological roles of miRNA–circRNA interactions may help to develop novel anti-cancer therapies that target circRNA-miRNA networks. Moreover, miR-141 and its circRNA sponges are associated with tumor growth and patients’ survival in some cancers, which makes them potential targets for diagnosis and therapy.

## Figures and Tables

**Table 2:** Summary of the interactions between circRNAs and miR-141 in different types of human cancer.

Cancer Type	circRNA	Upregulated miR-141-3p Targets	Biological Effects	References
**Breast cancer (BC)**	hsa_circ_0009362 (circGNB1)	IGF1R	promotes proliferation and migration of TNBC cells; increases tumor growth and lung metastases in mouse xenograft model	[94]
	hsa_circ_0075943	AK2	Knock-out of hsa_cicrc_0075943 suppresses the growth of cancer cells and induces apoptosis	[95]
**Lung adenocarcinoma (LUAD)**	hsa_circ_102442 (circKEAP1)	KEAP1	circKEAP1 sponges miR-141-3p which increases KEAP1 and decreases NRF2 expression and inhibits cancer growth	[96]
**Gastric cancer (GC)**	hsa_circ_0008583 (circDLG1)	CXCL12	induces infiltration of myeloid-derived suppressor cells to impair the function of CD8^+^ T cells and promotes GC progression; enhances GC cells proliferation, migration, invasion, EMT, immune evasion, and metastasis in immunocompetent mice	[97]
**Prostate cancer (PC)**	hsa_circ_0001206 (circCRKL)	KLF5	represses cell cycle, invasion, migration, stimulates apoptosis in vitro, and reduces tumor progression in vivo	[98]
	hsa_circ_0001633 (circSOBP)	MYPT1	inhibits migration and invasion of PC cells in vitro by the regulation of their ameboid migration and reduces metastasis in vivo	[99]
	hsa_circ_0004907 (circZEB1)	ZEB1	TR4 induced circZEB1 expression decreases PC radiosensitivity via TR4/QKI/circZEB1/miR-141-3p/ZEB1 axis	[100]
**Hepatocellular carcinoma (HCC)**	hsa_circ_100338	RHEB	hsa_circ_100338 activates mTOR signaling pathway and is associated with poor prognosis	[101,102]
	hsa_circ_100338	ZEB1	increases proliferation of HCC cells in vitro and in vivo	[103]
	hsa_circ_0008216 (circ-SMG1.72)	GSN	ERα suppresses circ-SMG1.72 and reduces HCC cell invasion via ERα/circ-SMG1.72/miR-141-3p/GSN axis	[104]
**Thyroid cancer (TC)**	hsa_circ_0015278 (circRNA_100395)		circRNA_100395 is downregulated in papillary thyroid carcinoma tissues compared to normal thyroid tissues and benign thyroid lesions	[105]
**Bladder Cancer (BlC)**	hsa_circ_0085361 (circTRPS1)	GLS1	exosome-derived circTRPS1 decreases intracellular reactive oxygen species level and induces CD8^+^ T cell exhaustion in the tumor microenvironment which promotes BlC growth and metastasis via circTRPS1/miR-141-3p/GLS1 axis	[106]
**Oral Squamous Cell Carcinoma (OSCC)**	hsa_circ_0072387		low hsa_circ_0072387 expression in OSCC tissues is associated with shorter overall survival of OSCC patients and related to higher TNM stage and tumor diameter	[107]
**Bone Cancer**	circ-LRP6	HDAC4, HMGB1	promotes proliferation, migration, and invasion of osteosarcoma cells	[108]

**Abbreviations:** AK2—adenylate kinase 2; CXCL12—C-X-C motif chemokine 12; ERα—estrogen receptor alpha; GLS1—glutaminase-1; GSN- gelsolin; HDAC4—histone deacetylase 4; HMGB1—high mobility group protein B1; IGF1R—insulin-like growth factor 1 receptor; KEAP1—Kelch-like ECH-associated protein 1; KLF5—Krüppel-like factor 5; mTOR—mechanistic target of rapamycin; MYPT1—protein phosphatase 1 regulatory subunit 12A; NRF2—nuclear factor erythroid 2–related factor 2; QKI—KH domain-containing RNA-binding protein QKI; RHEB—GTP-binding protein Rheb; TNBC—triple-negative breast cancer; TR4—testicular receptor 4; ZEB1—zinc finger E-box-binding homeobox 1.

## Data Availability

No new data were created and analyzed in this manuscript. Data sharing is not applicable.
